# DJ-1 as a Novel Therapeutic Target for Mitigating Myocardial Ischemia–Reperfusion Injury

**DOI:** 10.1155/cdr/6615720

**Published:** 2024-12-12

**Authors:** Jia-Bin Zhou, Tian-Peng Wei, Dan Wu, Feng Zhou, Ru-Xing Wang

**Affiliations:** Department of Cardiology, The Affiliated Wuxi People's Hospital of Nanjing Medical University, Wuxi People's Hospital, Wuxi Medical Center Nanjing Medical University, Wuxi 214023, China

**Keywords:** DJ-1, ischemia–reperfusion injury, myocardial, therapeutic target

## Abstract

Ischemic heart disease (IHD) remains one of the most prominent causes of mortality and morbidity globally, and the risk of ischemia–reperfusion injury is becoming more severe and constant. This underscores the need to develop new methods to protect the heart from damage. DJ-1 is a multifunctional intracellular protein encoded by the *PARK7* gene that plays roles in processes including the control of autophagy, the preservation of mitochondrial integrity, the prevention of apoptosis, and the elimination of oxidative stress. DJ-1 has recently been the focus of growing interest as a target molecule relevant to treating myocardial ischemia–reperfusion injury due to its protective properties and its role in cellular response mechanisms. Consistently, DJ-1-related interventions, such as its exogenous administration or the use of pharmacological agents, have been demonstrated to help protect the myocardium from ischemia–reperfusion injury and associated adverse outcomes. This review provides an overview of DJ-1 and its therapeutic relevance in the myocardium in the setting of ischemia and reperfusion.

## 1. Introduction

Myocardial ischemia–reperfusion injury (IRI) is a pressing cardiological issue worldwide, as it entails the exacerbation of existing tissue damage when blood flow is restored following an ischemic event. IRI is a common complication associated with coronary bypass grafting and percutaneous coronary intervention procedures, exposing patients to extensive morbidity including heart failure and myocardial infarction [1]. The pathophysiological basis for myocardial IRI entails complex interactions between processes including oxidative stress, inflammatory signaling, mitochondrial dysfunction, and calcium overload. Together, these processes culminate in the apoptotic and necrotic death of cardiomyocytes, ultimately impairing cardiac function and postinjury recovery [2].

DJ-1 (encoded by *PARK7*) was first identified as a protein associated with Parkinson's disease, but it has since been demonstrated to serve as a key cytoprotective factor in a variety of cellular contexts. It plays important roles in the induction of antioxidant stress responses, the maintenance of mitochondrial integrity, and the control of apoptotic signaling [3]. DJ-1 has recently been highlighted as a promising cardioprotective agent for the management of myocardial IRI owing to its ability to prevent inflammatory signaling and oxidative damage, ultimately leading to the better preservation of ischemic myocardial tissue functionality. The further elucidation of the mechanisms whereby DJ-1 can exert these protective effects in the context of myocardial IRI thus has the potential to inform the development of novel therapies capable of alleviating the burden of ischemic heart injury. This review offers a comprehensive overview summarizing current knowledge of the role that DJ-1 plays in the preservation of myocardial integrity, with a particular focus on its therapeutic value and future directions for research [4].

## 2. The Biological Characteristics of DJ-1

**Figure 1:**
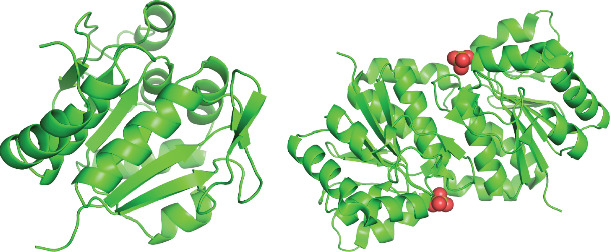
The crystal structure of DJ-1. (a) Spatial structure of protein DJ-1 from the UniProt database (ID: Q99497). (b) The dimerization configuration of DJ-1 (PDB: 2OR3). All the data above are from *Homo sapiens*.

DJ-1, existing in the UniProt database as Parkinson's disease protein 7 (Figure 1(a)), is a 189-amino-acid protein with a high degree of evolutionary conservation and atypical peroxiredoxin-like peroxidase activity that can protect against cellular stress through various mechanisms [5]. The three-dimensional (3D) structure of DJ-1 is characterized by a *β*-sheet flanked by *α*-helices, which is a common feature of proteins that play a role in shaping redox homeostasis. Through this configuration, DJ-1 is able to serve as a sensor for oxidative stress such that its protective functions are enhanced by structural changes stemming from the oxidative modification of cysteine residues within this protein [6]. In vivo, DJ-1 naturally forms a dimer (Figure 1(b)). Mutant proteins with Parkinson's disease–typical amino acid substitutions exhibit reduced dimerization, stability, and folding capacities. Mutations like L166P and M26I significantly hinder DJ-1 activity, with the L166P substitution being particularly detrimental. This mutation severely disrupts the protein's 3D structure and its ability to form homodimers [7].

DJ-1 contains three cysteine residues (C46, C53, and C106), with C106 being the most crucial for its function. C106 is highly sensitive to oxidative stress; its oxidation state directly correlates with DJ-1's activity levels, highlighting its essential role in the protein's functionality. Upon exposure to oxidative stress, the thiol group of C106 sequentially oxidizes to sulfinic (SOH), sulfonic (SO2H), and sulfonic (SO3H) acids. The C106A mutation obstructs the formation of these oxidized states, thus preventing DJ-1 from functioning effectively. Unlike the C106A mutation, similar substitutions at other cysteine residues, such as C46A or C53A, do not affect the protein in the same manner. Moreover, the C106A mutation inhibits DJ-1's translocation to mitochondria [8]. While the oxidation of C106 has minimal impact on DJ-1's overall structure, it significantly reduces the aggregation of *α*-synuclein. However, profound oxidation results in changes to the protein's secondary structure and diminishes its ability to inhibit *α*-synuclein aggregation [9].

Ubiquitous DJ-1 expression has been reported across cell and tissue types, and it predominantly localizes to the cytoplasm within cells. However, mitochondrial, nuclear, and extracellular DJ-1 translocation has been reported in response to stress and altered cellular homeostasis. This process of dynamic localization highlights the role that DJ-1 can play as a regulator of diverse processes including mitochondrial quality control and the regulation of transcription [10].

The expression of DJ-1 is tightly regulated both transcriptionally and posttranscriptionally. Oxidative stress can induce DJ-1 transcriptional upregulation in response to the activation of the clear factor erythropoiesis–related factor 2 (NRF2) and PI3K/Akt signaling pathways, for example, allowing cells to better defend against adverse conditions. The stability and activity of DJ-1 can also be influenced by posttranslational acetylation and phosphorylation, enabling fine-tuned control of its cellular functionality [11].

DJ-1 is a highly versatile protein, as evidenced by its ability to interact with a variety of proteins and to play a role in many distinct biochemical pathways. Its intracellular protective functions are not solely attributable to its peroxidase-like activity, as it can also serve as a glyoxalase that detoxifies cytotoxic glycolysis byproducts, controls mitochondrial dynamics, and serves as a molecular chaperone [12]. Diminished functionality of reduced DJ-1 contributes to the pathogenesis of oxidative stress–related diseases such as Parkinson's disease, Type II diabetes, and male infertility. Conversely, hyperactivation of DJ-1 is implicated in the development of cancer [13]. The broad functional and regulatory spectrum that characterizes DJ-1 is integral to its status as a central regulator of cellular stress responses and survival, highlighting its promise as a target for therapeutic intervention in the management of myocardial IRI and other pathological conditions related to stress.

## 3. The Pathophysiological Basis for Myocardial Ischemia–Reperfusion Injury

The etiology of myocardial ischemia–reperfusion injury is complex. It begins with the disruption of the cardiac blood supply and is followed by its restoration. These two events trigger a series of deleterious cellular and biochemical responses that contribute to the exacerbation of the cardiac damage at levels exceeding those solely attributable to the initial ischemia [14].

During the initial ischemic phase, initial reductions in blood supply result in severe oxygen and nutrient deprivation. Under the resultant hypoxic conditions, cardiomyocytes must undergo a metabolic shift away from aerobic metabolism in favor of anaerobic metabolism, leading to lower levels of ATP production and the accumulation of high levels of lactate and other metabolic byproducts. Importantly, such ischemia also leads to the impairment of mitochondrial functionality, disrupting electron transport and contributing to reactive oxygen species (ROS) formation. The absence of sufficient oxygen and nutrients also leads to the destabilization of cellular membranes as a consequence of ATP depletion, given its necessity for the function of ion pumps, ultimately culminating in cellular edema and necrosis [2].

When reperfusion occurs, the rapid influx of oxygenated blood can, seemingly counterintuitively, generate high levels of ROS that drive an increase in tissue damage. These free radicals can rapidly exceed the capacity of the cellular antioxidant system, imposing conditions of oxidative stress. Such stress, in turn, results in additional mitochondrial DNA and protein damage, leading to even more severe energy insufficiency and the enhanced engagement of the apoptotic and necrotic cell death pathways [15].

Reperfusion can also trigger high levels of inflammation that are characterized by extensive neutrophilic infiltration and activation in the myocardium, with similar responses for other immune cell types. These cells can then secrete a wide range of proinflammatory chemokines, cytokines, and other enzymes capable of exacerbating local tissue injury through increases in vascular permeability while also promoting higher levels of leukocyte recruitment and activation, fueling a vicious cycle of inflammation and damage [16].

In conclusion, the pathophysiology of myocardial IRI is characterized by interactions between oxidative stress, cell damage, and inflammation after reperfusion. Each of these components can give rise to impaired cellular function after myocardial infarction, underscoring the importance of targeted therapies capable of mitigating these damaging effects.

## 4. The Plausible Mechanisms Through Which DJ-1 Protects Against Myocardial IRI

### 4.1. Antioxidant Activity

DJ-1 is a protein that is broadly expressed, and its cytoprotective activity is tied in large part to its ability to counteract oxidative stress. Under oxidative stress conditions, the thiol residues in DJ-1 can become oxidized, thereby modulating the activity of the protein as a whole. DJ-1 is also capable of stabilizing other proteins with antioxidant activity including NRF2, bolstering the overall cellular antioxidant capacity. DJ-1 enhances NRF2 stability and translocation within the nucleus by shielding it from degradation mediated by Keap1. The activation of NRF2 results in the upregulation of key antioxidant enzymes including SOD, catalase (CAT), and glutathione peroxidase (GPX), rendering cardiomyocytes better able to tolerate conditions of oxidative stress. These processes lead to reductions in the ROS levels generated under ischemia/reperfusion conditions while also attenuating cellular damage.

In rat models of myocardial IRI, DJ-1 has been demonstrated to offer potent cardioprotective antioxidant activity [17]. Yan et al. [18] further determined that NRF2 pathway activation and consequent antioxidant enzyme upregulation may be the primary mechanism through which DJ-1 can serve as a mediator of the delayed cardioprotective efficacy of hypoxic preconditioning, enabling cardiomyocytes to resist the effects of hypoxia and reoxygenation. DJ-1 harbors three cysteine residues at positions 46, 53, and 106, with cysteine 106 exhibiting the greatest potential for oxidation [9]. Liu et al. [19] demonstrated the ability of oxidized DJ-1 (C106) to interact with PTEN, negatively regulating its phosphatase activity while activating the p-IKK/NF-*κ*b/Beclin1 signaling pathway to aggravate myocardial IRI. In models of myocardial IRI, DJ-1 can suppress ROS-induced apoptosis while also preserving mitochondrial function through the suppression of mitochondrial ROS biogenesis and the maintenance of mitochondrial potential, thereby altering autophagic activity and mitochondrial biogenesis, protecting cardiomyocytes against injury. DJ-1 is thus a key antioxidant protein that can help protect against myocardial IRI through its effects on several different mechanistic pathways.

### 4.2. Antiapoptotic Activity

The nicotinamide adenine dinucleotide (NAD+)–dependent Class III histone deacetylase Sirtuin 1 (SIRT1) can help protect cardiac tissue against the effects of IRI [20]. Xu et al. [21] developed cell-based models of ischemia–reperfusion that they then treated with SIRT1 inhibitors and DJ-1-specific siRNA constructs, ultimately demonstrating that DJ-1 is capable of directly binding SIRT1 and stimulating its deacetylase activity, ultimately driving the activity of the SIRT1 target p53 to reduce the apoptotic death of cardiomyocytes. DJ-1 has been demonstrated to help support the survival of cardiomyocytes through its ability to inhibit PTEN and activate Akt (protein kinase B) [22]. Li et al. [23] determined that knocking down DJ-1 led to the enhancement of cardiomyocyte and myocardial tissue apoptosis rates in vivo and ex vivo when using experimental rats. Specifically, suppressing DJ-1 expression led to increases in the levels of the apoptosis-related Bax and Cleaved Caspase-3 proteins together with the activation of PTEN/Akt signaling. N-Acetylcysteine administration drove the upregulation of DJ-1 and the attenuation of diabetic myocardial IRI through the suppression of PTEN/Akt signaling. Daxx is an established regulator of apoptotic death in cardiomyocytes such that overexpressing Daxx can protect against apoptosis, whereas its downregulation increases apoptotic sensitivity [24]. DJ-1 has previously been identified as an inhibitor of apoptosis owing to its ability to sequester Daxx within the nucleus, preventing its cytoplasmic entry such that it is unable to bind to or activate the downstream effector kinase Apoptosis Signal–Regulating Kinase 1 (ASK1) [25]. Zobalova et al. [24], however, posited that DJ-1 lacks this nuclear sequestration–mediated ability to modulate the apoptotic regulatory activity of Daxx in cardiomyocytes, highlighting a need for additional experimental validation.

### 4.3. Regulation of Autophagy

Autophagy is a conserved process through which cells can remove damaged proteins and organelles through lysosomal degradation [26]. Autophagy plays a vital role in the maintenance of cellular homeostasis, particularly under conditions of stress. Autophagy plays a dual role in cardiac health: During the ischemic phase, its activation facilitates the removal of excess metabolic waste, promoting cardiomyocyte survival. However, during reperfusion, excessive autophagy can deplete essential cellular components, resulting in autophagic cell death [27]. In the context of cardiac hypertrophy, knocking down DJ-1 can significantly inhibit cardiomyocyte autophagy and enhance TORC 1 and mTORC2 phosphorylation, while overexpressing DJ-1 can alleviate phenylephrine-induced cardiac hypertrophy and promote autophagy within cardiomyocytes [28]. Research indicates that among the three cysteine residues in DJ-1 (C46, C53, and C106), mutation at C106 not only diminishes DJ-1's protective effects but also impairs its ability to bind with other proteins [29]. In a model of IRI, Zhao et al. demonstrated that the protein DJ-1 interacts with RACK1 to form a complex that initiates adaptive autophagy via the adenosine 5⁣′-monophosphate–activated protein kinase (AMPK)/mechanistic target of rapamycin (mTOR) signaling pathway. This interaction ultimately confers protection to the myocardium against ischemia/hypoxia damage. Conversely, a mutation at the Cysteine Residue C106 in DJ-1 disrupts the DJ-1-RACK1 complex formation and impedes the protective autophagy mediated by DJ-1 [30]. Liu et al. [31] demonstrated that resveratrol significantly enhances DJ-1 expression in the myocardium following IRI. Additionally, they found that resveratrol mitigates excessive autophagy in myocardial IRI by reducing the phosphorylation of MAPK/ERK Kinase Kinase 1 (MEKK1) and Jun N-terminal kinase (JNK) through the upregulation of DJ-1 in both in vivo and in vitro studies. Importantly, the autophagy promoter rapamycin was shown to negate the cardioprotective effects of resveratrol. Research has indicated that DJ-1 can directly bind to and activate SIRT1, a member of the sirtuin family of proteins that is linked to longevity and antiaging. DJ-1 enhances the deacetylase activity of SIRT1 [13]. Furthermore, resveratrol is known to promote adaptive autophagy through activation of SIRT1 [32]. Thus, the upregulation of SIRT1 by resveratrol may be intricately associated with the activation of DJ-1.

### 4.4. Mitochondrial Protection

The impairment of mitochondrial function can profoundly affect the recovery process following myocardial ischemia–reperfusion, with the degree of ischemia–reperfusion-induced mitochondrial dysfunction thus being closely related to the extent of myocardial injury and cardiomyocyte death. DJ-1 has recently been established as an endogenous protein with cardioprotective activity, and DJ-1 can protect against cytotoxicity in settings of acute IRI through the prevention of mitochondrial dysfunction [4]. Shimizu et al. [33] further found that myocardial IRI-induced DJ-1 activation was sufficient to protect the heart through the regulation of DRP1 SUMO status and the attenuation of overly high levels of mitochondrial fission. DJ-1 has also been found to attenuate the postischemic glycation of cardiac Mitochondrial Complex I and Complex III, thereby helping to maintain their efficiency and overall mitochondrial function while recovering from IRI [34]. In H9C2 cardiomyocytes, resveratrol has been shown to help prevent hypoxia-/reoxygenation-induced injury through its ability to promote mitochondrial DJ-1 translocation and the maintenance of Mitochondrial Complex I activity [35]. Zhou et al. [36] additionally found that Grp75 is capable of interacting with DJ-1 to promote its translocation from the cytoplasm to the mitochondria, with such activity being essential for the resveratrol-mediated preservation of Mitochondrial Complex I activity and for protection against myocardial IRI. These results thus offer further evidence in support of developing therapeutic approaches targeting DJ-1.  Table 1 summarizes examples illustrating DJ-1 interaction with its protein partners.

Table 1 summarizes examples illustrating DJ-1 interaction with its protein partners.

## 5. Clinical Prospects and Challenges

There has been growing interest in the application of DJ-1 as a target or tool for the treatment of myocardial IRI. Efforts to translate DJ-1-related research findings into clinical therapies primarily focus on its antioxidant capacity and ability to control mitochondrial integrity, suggesting that it may help attenuate key IRI-related pathological processes.

Gallinat et al. [37] determined that exogenously administering DJ-1 could provide protection against myocardial infarction while also modulating cellular activity associated with cardiac injury in vivo. Resveratrol, as a natural polyphenol, has been the focus of extensive interest owing to its cardioprotective properties. Resveratrol is capable of modulating MEKK1/JNK signaling activity via DJ-1 to suppress autophagy in the context of myocardial IRI [31]. It can additionally promote DJ-1 upregulation, activate SIRT1, and inhibit p53 acetylation to suppress IRI-induced apoptotic cardiomyocyte death [21]. Efforts to design DJ-1-based therapeutic strategies may entail gene therapy efforts focused on enhancing DJ-1 expression within cardiac tissues or utilizing small molecules to enhance the protective benefits of this protein.

While DJ-1 thus exhibits an array of promising clinical features, there are some persistent barriers to its clinical application. For one, the precise mechanisms that DJ-1 uses to exert its cardioprotective effects have not been fully documented, and additional research is thus vital at both the cellular and molecular levels. Secondly, specifically delivering DJ-1 or activators thereof to the heart remains difficult, and current delivery strategies are hampered by the potential for off-target effects and toxicity. Additionally, the specific timing of therapeutic interventions is of critical importance, as IRI-related damage can only be effectively mitigated within a narrow therapeutic window.

Future investigations should seek to explore the signaling pathways in cardiac cells that involve DJ-1 at greater length, as these efforts will inform the development of precisely targeted drugs that can modulate its functionality. Designing advanced delivery systems capable of selectively targeting cardiac cells, such as nanoparticle-based carriers, is also important. Studies examining interactions between DJ-1 and other cytoprotective drugs may also help guide the design of synergistic therapies capable of more effectively mitigating the effects of myocardial IRI.

In summary, DJ-1 holds promise as a target for the attenuation of myocardial IRI. However, fully realizing its clinical potential will require concerted efforts to overcome challenges related to administration, timing, and a detailed understanding of the underlying mechanisms of action.

## 6. Conclusion

**Figure 2:**
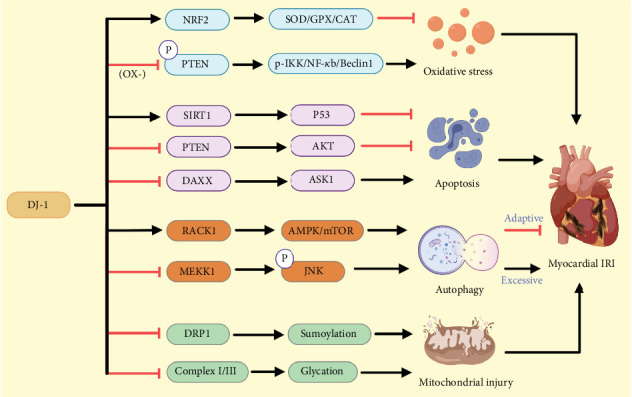
Regulatory mechanism of DJ-1 in myocardial ischemia–reperfusion injury (IRI). Myocardial IRI is characterized by additional myocardial damage resulting from the restoration of blood flow, a process entailing complex biochemical and physiological mechanisms. This type of injury can precipitate a further decline in cardiac function. DJ-1, a multifunctional intracellular protein, engages in various protective responses. In the context of myocardial ischemia–reperfusion, DJ-1 mitigates oxidative stress by enhancing NRF2 activity and suppressing PTEN phosphorylation. Additionally, DJ-1 exerts antiapoptotic effects by upregulating SIRT1 expression and inhibiting the PTEN and Daxx pathways. DJ-1 also promotes adaptive autophagy through interaction with RACK1 and prevents excessive autophagy by inhibiting the MEKK1 pathway. Moreover, DJ-1 safeguards mitochondria from damage by blocking the sumoylation of DRP1 and moderating the glycolytic activity of Mitochondrial Complexes I and III. Blunt-ended lines indicate inhibition while arrows indicate promotion. IRI, ischemia–reperfusion injury; NRF2, Nuclear Factor Erythroid 2–Related Factor 2; SOD, superoxide dismutase; GPX, glutathione peroxidase; CAT, catalase; PTEN, phosphatase and tensin homolog; IKK, I*κ*B kinase; NF-*κ*b, nuclear factor kappa-light-chain-enhancer of activated B cells; SIRT1, Sirtuin 1; AKT, Ak strain transforming; Daxx, death domain–associated protein; ASK1, Apoptosis Signal–Regulating Kinase 1; RACK1, Receptor for Activated C Kinase 1; AMPK, adenosine 5⁣′-monophosphate (AMP)–activated protein kinase; mTOR, mechanistic target of rapamycin; MEKK1, MAP/ERK Kinase Kinase 1; JNK, c-Jun N-terminal kinase; DRP1, Dynamin-Related Protein 1.

In summary, this article provides an overview of the key mechanisms through which DJ-1 exerts its protective effects in the context of myocardial IRI while also discussing the utilization of DJ-1-related drugs for the management of this debilitating cardiac condition (Figure 2). Given that ischemic heart disease remains the leading cause of disability and death globally, it is essential that the pathophysiology of myocardial IRI be fully elucidated and new therapeutic targets be identified. DJ-1 is a ubiquitously expressed multifunctional redox-responsive protein that is involved in the suppression of oxidative stress, the preservation of mitochondrial integrity, the control of autophagy, and the prevention of apoptosis. Future studies will help cement the status of DJ-1 as an invaluable cardioprotective factor for the management of myocardial IRI.

## Figures and Tables

**Table 1:** Interaction of DJ-1 protein with its protein partners.

**DJ-1 protein partners**	**Experimental species**	**Biological effect**	**References**
Nuclear Factor Erythroid 2–Related Factor 2 (NRF2)	*Rattus norvegicus*	DJ-1 enhances NRF2 stability and translocation within the nucleus by shielding it from degradation mediated by Keap1	[17, 18]
Phosphatase and tensin homolog (PTEN)	*Rattus norvegicus*	The ability of oxidized DJ-1 (C106) to interact with PTEN, negatively regulating its phosphatase activity while activating the p-IKK/NF-*κ*b/Beclin1 signaling pathway	[19]
	*Homo sapiens* *Rattus norvegicus*	DJ-1 supports the survival of cardiomyocytes through its ability to inhibit PTEN and activate AKT	[22] [23]
Sirtuin 1 (SIRT1)	*Rattus norvegicus*	DJ-1 is capable of directly binding SIRT1 and stimulating its deacetylase activity, ultimately driving the activity of the SIRT1 target p53 to reduce the apoptotic death of cardiomyocytes	[20, 21]
Death domain–associated protein (Daxx)	*Rattus norvegicus* *Homo sapiens*	DJ-1 modulates Daxx and activates its downstream effector kinase, Apoptosis Signal–Regulating Kinase 1 (ASK1)	[24] [25]
Receptor for Activated C Kinase 1 (RACK1)	*Rattus norvegicus*	DJ-1 interacts with RACK1 to form a complex that initiates adaptive autophagy via the AMPK/mTOR signaling pathway	[30]
MAP/ERK Kinase Kinase 1 (MEKK1)	*Rattus norvegicus*	DJ-1 reduces phosphorylation of MAPK/ERK Kinase Kinase 1 (MEKK1) and Jun N-terminal kinase (JNK), thereby inhibiting excessive autophagy	[31]
Dynamin-Related Protein 1 (DRP1)	*Mus musculus*	DJ-1 is sufficient to protect the heart through the regulation of DRP1 SUMO status and the attenuation of overly high levels of mitochondrial fission	[33]
Mitochondrial Complexes I and III	*Mus musculus* *Rattus norvegicus*	DJ-1 can attenuate the postischemic glycation of cardiac Mitochondrial Complexes I and III to maintain their efficiency and mitochondrial function	[34, 35] [36]

## Data Availability

The authors have nothing to report.
